# Natural killer cell-specific chimeric antigen receptor enhances CAR NK cell functions and anti-tumor activity

**DOI:** 10.7150/thno.120909

**Published:** 2025-11-06

**Authors:** Changqing Pan, You Zhai, Zhongliang Cui, Yiyun Yin, Menghui Xu, Di Wang, Yishuo Sun, Jiazheng Zhang, Chen Wang, Ziwei Li, Mingchen Yu, Zhongfang Shi, Guanzhang Li, Tao Jiang, Wei Zhang

**Affiliations:** 1 Department of Neurosurgery, Beijing Tiantan Hospital, Capital Medical University, Beijing, PR China.; 2 Department of Molecular Neuropathology, Beijing Neurosurgical Institute, Capital Medical University, Beijing, PR China.; 3 Department of Pathophysiology, Beijing Neurosurgical Institute, Capital Medical University, Beijing, PR China.; 4 Brain Tumor Center, Beijing Institute of Brain Disorders, Capital Medical University, Beijing 100070, PR China; 5 Beijing Engineering Research Center of Targeted Drugs and Cell Therapy for CNS Tumors, Beijing 102600, PR China.; 6 China National Clinical Research Center for Neurological Diseases, Beijing, PR China.; 7 National Center for Neurological Disorders, Beijing 100070, PR China.; 8 Chinese Glioma Genome Atlas Network (CGGA) and Asian Glioma Genome Atlas Network (AGGA), Beijing, PR China.

**Keywords:** CAR NK, NK-92MI, iPSC, glioblastoma, lymphoma

## Abstract

**Background**: Unlike T cells, natural killer (NK) cells lack a dominant activating receptor analogous to the T cell receptor (TCR) that governs their activation. Whether chimeric antigen receptor (CAR) constructs engineered specifically for T cells can effectively drive NK cell activation remains unresolved. NK cells inherently possess non-specific recognition capacities and exert broad-spectrum cytotoxicity against diverse tumor targets. However, the complexity of receptor-ligand interactions between CAR NK cells and susceptible target cells has impeded efforts to delineate the specific functional contributions of individual CAR constructs.

**Methods**: CAR NK cells were generated via electroporation. The murine B16 melanoma cell line was modified to express various target proteins using lentiviral transduction. *In vitro* functional assays, including conjugate formation, granule polarization, degranulation, cytotoxicity, and cytokine production, were employed to assess CAR NK cell efficacy. Recombinant protein-coated beads were used to investigate downstream activation signaling pathways. The *in vivo* antitumor activity of CAR NK cells was evaluated using NPG mouse xenograft models.

**Results**: B16 cell line was first validated to be a suitable model for specifically assessing CAR construct function in CAR NK cells. Among nine distinct CAR molecules generated, the construct incorporating the NKG2DTM-2B4-FCER1G exhibited the most potent capacity to enhance NK cell-mediated functionalities. Consistent with these functional improvements, this CAR construct induced robust phosphorylation of key activation pathways, including AKT, VAV1, ERK, PLCγ1, and NF-κB.

**Conclusions**: The CAR construct incorporating the NKG2DTM-2B4-FCER1G is demonstrated to be the most effective in enhancing NK cell functionality.

## Introduction

CAR T immunotherapy has demonstrated remarkable efficacy in treating B-cell malignancies and autoimmune diseases 1, 2. However, autologous CAR T cell therapy is hampered by inherent limitations, including prolonged manufacturing timelines, high costs, and life-threatening toxicities 3, which fueled interest in alternative therapeutic strategies. NK cells, a key subset of the innate immune system, are critical for immune surveillance. A growing body of clinical evidence has highlighted the promising antitumor activity and favorable safety profile of allogeneic CAR NK cells 4-7.

CAR constructs, designed for T cells (incorporating CD28 or 4-1BB as costimulatory domain and CD3ζ as signaling domain), remain the most widely used for generating CAR NK cells 8. However, T cells and NK cells exhibit distinct activation paradigms. The two-signal activation theory in T cells, where the first signal is delivered by antigen peptide-MHC complexes and the second by costimulatory molecules such as CD28, is currently the most widely accepted 9. Unlike T cells, natural killer (NK) cells lack a single activating receptor like TCR that dominates their activation, which raises questions about the suitability of T cell-specific CAR designs for NK cell activation. This uncertainty has spurred increasing interest in developing NK cell-specific CAR constructs 1, 10, 11.

NK cells express a diverse repertoire of germline-encoded receptors 12. Among these, FcγRIIIA (CD16) is the only activating receptor identified to induce degranulation in resting NK cells 13. CD16 transduces activation signals via two adaptor molecules, the γ chain (FCER1G) or CD3ζ 14, 15. It can associate with ζζ, γγ, or ζγ dimers, with a preference for γγ homodimers 16. A prior study demonstrated that a CAR containing the FCER1G cytoplasmic tail induced more efficient cytotoxicity than one with CD3ζ 17, suggesting FCER1G may be a superior signaling domain for CAR NK cells.

Synergistic combinations of coactivating receptors are usually required to activate NK cells 13. The 2B4 receptor has been shown to enhance CD16-dependent degranulation and cytokine production 18, 19, while NKG2D also acts as a potent co-stimulator of CD16-mediated degranulation 20. Co-engagement of 2B4 more effectively amplifies CD16-mediated signaling for degranulation compared to NKG2D 21. A previous study has identified a CAR incorporating the NKG2D transmembrane domain, 2B4 intracellular co-stimulatory domain, and CD3ζ signaling domain that mediated robust antigen-specific NK cell signaling 10, supporting 2B4 as a promising coactivation domain for CAR NK cells.

NK cells exhibit non-specific recognition and broad-spectrum cytotoxicity against tumor targets, enabling CAR NK cells to kill targets via both CAR-dependent and CAR-independent mechanisms. However, the complexity of receptor-ligand interactions between CAR NK cells and sensitive target cells has hindered efforts to delineate the specific functions of individual CAR constructs. Drosophila insect cells, which lack ligands for human NK cell adhesion and activation receptors, have been widely used to dissect the contributions of individual NK cell receptors in previous studies 18, 19, 21. In the present study, we validated the murine B16 melanoma cell line as a robust model for evaluating the specific functions of CAR constructs to CAR NK. Using this model, we systematically assessed a range of coactivation domains (2B4 and DAP10) and signaling domains (FCER1G and CD3ζ) to identify the most potent CAR construct. Our findings reveal that the CAR construct incorporating the NKG2D transmembrane domain, 2B4 intracellular domain, and FCER1G signaling domain was the most efficient among the 9 CAR constructs. Mechanistically, the construct enhanced antigen-specific NK cell signaling by activating multiple downstream cascades, including AKT, VAV1, ERK, PLC-γ1, and NF-κB pathways. In both solid and hematologic tumor models, NK cells expressing this CAR construct delayed tumor growth and improved animal survival.

## Results

### The B16 cell line enables specific evaluation of CAR NK cell activity

**Figure 1:**
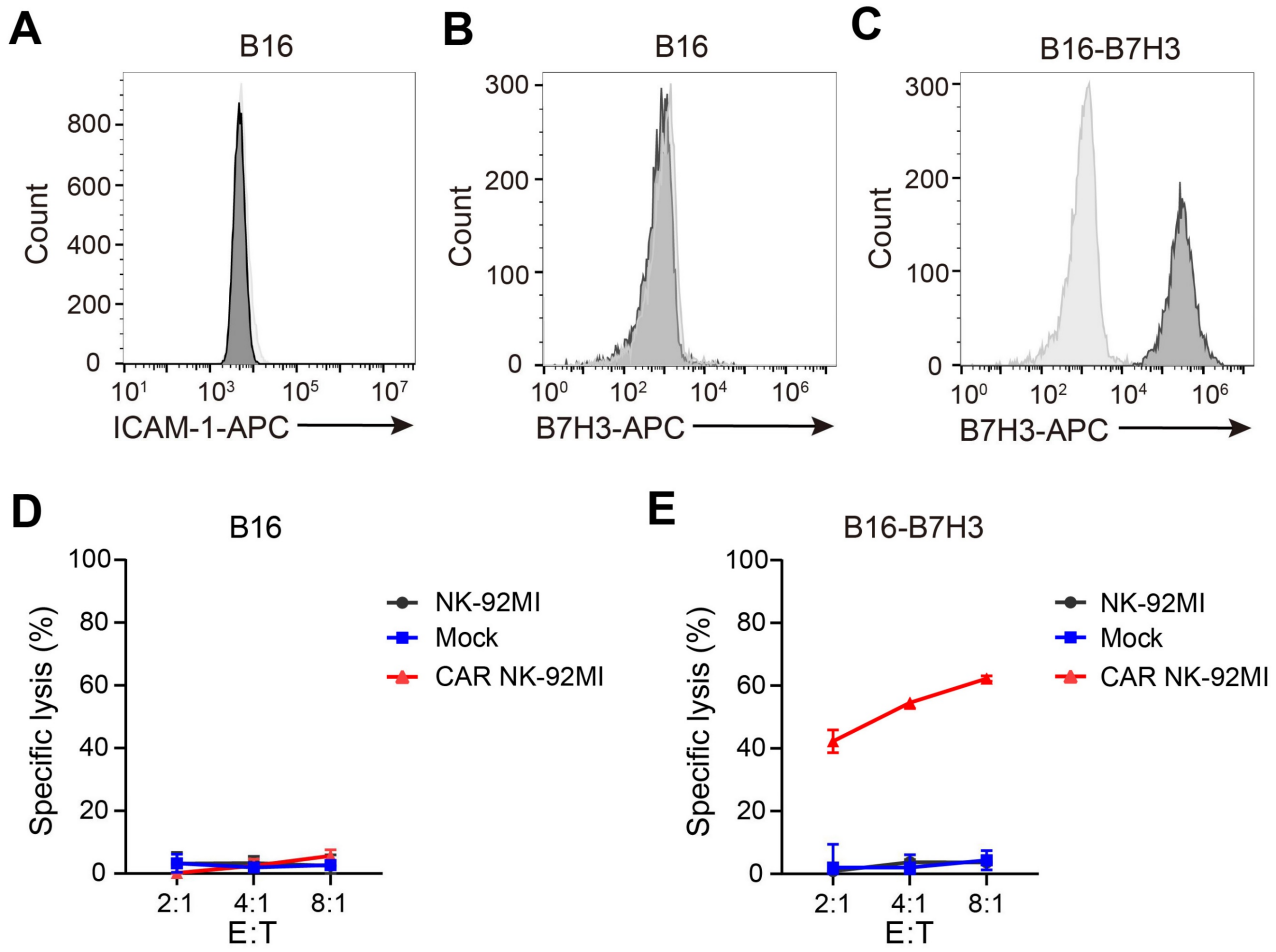
** The B16 cell line serves as a robust model for specific evaluation of CAR NK-92MI cell activity.** (**A-B**) Surface expression of ICAM-1 (**A**) and B7H3 (**B**) in B16 cells assessed by flow cytometry. (**C**) Generation of a B16 cell line overexpressing B7H3 via lentiviral transduction. (**D-E**) Representative specific cytotoxicity of Mock and CAR NK-92MI cells against B16 (**D**) or B16-B7H3 (**E**) cells at various effector-to-target ratios (n = 4). The CAR construct targets B7H3 and comprises the NKG2D transmembrane domain, CD28 intracellular domain, and CD3ζ intracellular signaling domain.

Intercellular adhesion molecule 1 (ICAM-1) is the only known murine activating ligand recognized by human NK cells 22. The murine B16 melanoma cell line was confirmed to lack ICAM-1 expression (Figure 1A) and did not express human B7H3 (Figure 1B). To assess the cytotoxicity of B7H3-targeting CAR NK cells, a B16 derivative overexpressing B7H3 (B16-B7H3) was generated via lentiviral transduction (Figure 1C). Cytotoxicity assays demonstrated that neither parental NK-92MI nor CAR-engineered NK-92MI cells killed B16 cells (Figure 1D), whereas B16-B7H3 cells were specifically lysed by CAR NK-92MI cells (Figure 1E). These findings validated the B16 cell line as a robust model for specifically evaluating CAR construct function to CAR NK cells.

### Generation and *in vitro* screening of NK cell-specific CAR constructs

**Figure 2:**
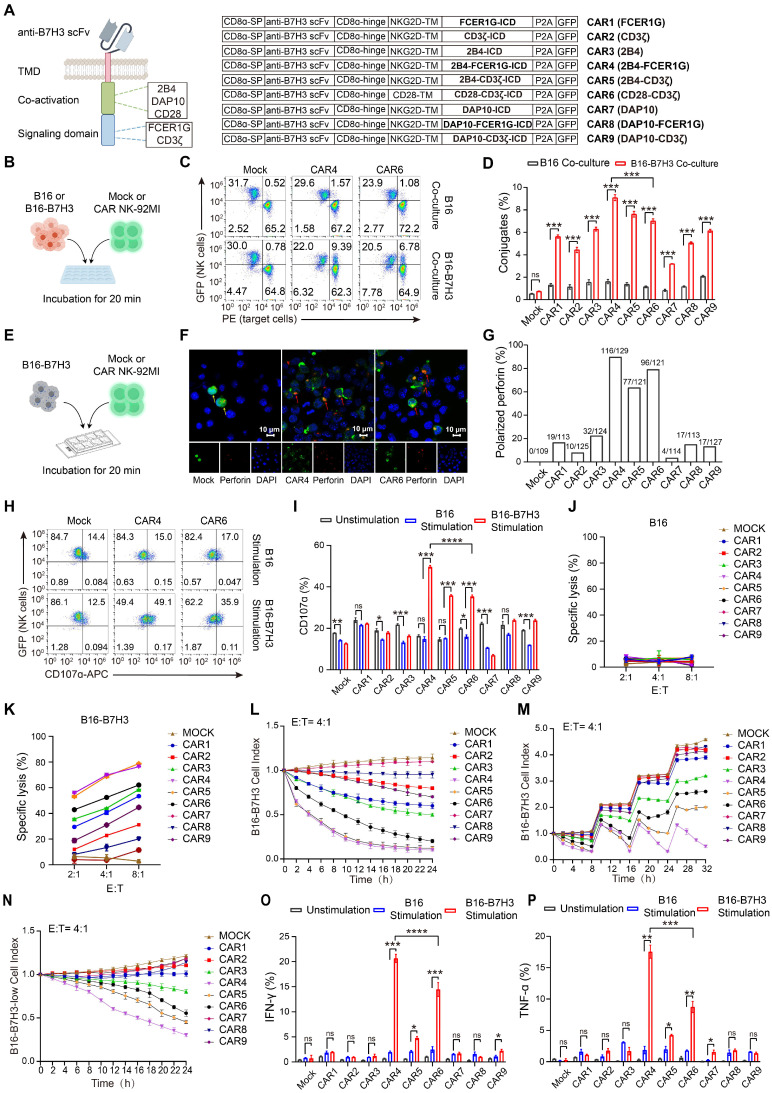
** Generation and *in vitro* characterization of NK cell-specific CAR constructs in NK-92MI cells.** (**A**) Schematic representation of B7H3-targeting CAR constructs. TMD, transmembrane domain. (**B**) Schematic of *in vitro* conjugate formation assays. B16 or B16-B7H3 cells labeled with orange fluorescence (PE channel) were co-incubated with GFP-expressing Mock or CAR NK-92MI cells for 20 min in duplicate. Mock NK-92MI cells were generated using the same vector backbone and expressed only GFP. (**C**) Representative flow cytometry plots showing conjugate formation between Mock, CAR4, or CAR6 NK-92MI cells and target cells. NK cell-target cell conjugates are identified as PE⁺GFP⁺ cells (upper right quadrant). (**D**) Summary of conjugate formation (n = 3). (**E**) Schematic of *in vitro* granule polarization assays. (**F**) Representative confocal microscopy images of GFP-expressing Mock, CAR4, or CAR6 NK-92MI cells co-cultured with B16-B7H3 cells, stained with Alexa 594-conjugated anti-perforin monoclonal antibody (60 × oil immersion objective). Perforin-containing granules were considered polarized when most fluorescence (randomly distributed in unstimulated cells) concentrated in the NK-92MI cell quadrant adjacent to the target. Red arrows indicate polarized granules; yellow arrows indicate non-polarized granules. (**G**) Summary of granule polarization, more than 100 NK-92MI cells per group were scored with a 40× objective to calculate the percentage of polarization. (**H**) Representative flow cytometry plots of degranulation (assessed by CD107α expression) in Mock, CAR4, or CAR6 NK-92MI cells against B16 or B16-B7H3 target cells. (**I**) Summary of degranulation (n = 3). (**J-K**) Representative specific killing of B16 (**J**) or B16-B7H3 (**K**) cells by Mock and CAR NK-92MI cells at different cell-to-cell ratios (n = 4). (**L**) Continuous killing assay: CAR NK-92MI cells (effector-to-target ratio 4 :1) were co-cultured with mCherry-expressing B16-B7H3 cells, monitored over 24 h using the Cellcyte X imaging system. Red fluorescence intensity was normalized to initial scans to calculate cell index. Values represent mean ± s.d. (n = 4 technical replicates per group). (**M**) Sequential killing assay: CAR NK-92MI cells were co-cultured with B16-B7H3 cells, with fresh target cells added every 8 h. Values represent mean ± s.d. (n = 4 technical replicates per group). (**N**) Cytolytic activity of CAR-NK-92MI cells against B16-B7H3-low target cells. Values represent mean ± SD (n = 4 technical replicates per group). (**O-P**) Summary of intracellular IFN-γ (**O**) and TNF-α (**P**) expression (n = 3). Data are presented as mean ± s.d. Statistical analyses were performed using two-way ANOVA followed by Sidak's (**D**) or Tukey's (**I**, **O**, **P**) multiple comparisons test.

We designed and screened 9 NK cell-optimized CAR constructs (designated CAR1-9; Figure 2A), all targeting B7H3 and co-expressing green fluorescent protein (GFP) via P2A peptide cleavage. CAR6, incorporating CD28 and CD3ζ, served as a control. The remaining NK cell-specific constructs consisted of the NKG2D transmembrane domain, either 2B4 or DAP10 as the coactivation domain, and either FCER1G or CD3ζ as the intracellular signaling domain. NK-92MI cells were electroporated with these CAR constructs, achieving comparable transduction efficiencies across all groups (Figure S1A). Mock NK-92MI cells were generated using the same vector backbone, expressing only GFP.

Modification with CAR constructs did not alter the expression of endogenous activating receptors in NK-92MI cells (Figure S1B). Intracellular flow cytometry revealed that perforin and granzyme levels in CAR NK-92MI cells were comparable to those in unmodified cells (Figure S2A). Upon stimulation with PMA and ionomycin, all NK-92MI cell lines exhibited reduced cytoplasmic perforin and granzyme (Figure S2B-D), accompanied by efficient and comparable degranulation (Figure S2E-F). Cell cycle analysis showed similar proliferative kinetics across all NK-92MI lines (Figure S2G-H), with all maintaining rapid proliferation rates (Figure S2I) and no significant differences in apoptosis (Figure S2J-K). These results confirmed that CAR modification does not impair the intrinsic cellular functions of NK-92MI cells.

Compared to B16 cells, all CAR NK-92MI groups except CAR2 and CAR7 exhibited significant activation (as indicated by upregulated CD69) upon exposure to B16-B7H3 cells, with CAR4 inducing the strongest activation (Figure S3). Given that conjugate formation between NK cells and targets is critical for early activation 23, 24, we assessed this interaction (Figure 2B). CAR NK-92MI cells formed more conjugates with B16-B7H3 cells than mock NK-92MI cells (Figure 2C-D; Figure S4A), with CAR4 NK-92MI cells showing the highest frequency. After contact with NK-sensitive target cells, lytic granule polarization toward targets is essential for NK cell cytotoxicity 20, 25. Thus, we evaluated granule polarization of CAR NK-92MI cells against B16-B7H3 cells (Figure 2E). In the absence of stimulation, perforin-containing granules were randomly distributed in both mock and CAR NK-92MI cells (Figure S4B). Upon co-culture with B16-B7H3 cells, CAR NK-92MI cells exhibited varying degrees of granule polarization at the cell-target interface (Figure 2F-G; Figure S4C), with CAR4 NK-92MI cells showing the highest polarization rate. Polarization of cytolytic granules at an early stage of conjugate formation may be a way to predispose NK cells to kill by degranulation as soon as other activation receptors are engaged. To this end, we performed degranulation assays by CD107a expression. Baseline degranulation was similar across all NK-92MI lines (15-25%) and unresponsive to B16 stimulation (Figure S4D). In contrast, CAR4 NK-92MI cells displayed significantly higher CD107a levels upon B16-B7H3 stimulation (Figure 2H-I; Figure S4E).

### *In vitro* cytotoxicity and cytokine production of NK cell-specific CAR constructs

Tumor cell killing assays confirmed that neither mock nor CAR NK-92MI cells killed B16 cells (Figure 2J), whereas CAR NK-92MI cells specifically lysed B16-B7H3 cells. Among these, CAR4 and CAR5 NK-92MI cells exhibited the highest cytotoxicity (Figure 2K), corresponding to increased granzyme secretion (Figure S5A-B). Continuous killing assays revealed that CAR4 and CAR5 NK-92MI cells maintained the highest and most sustained cytolytic activity against targets (Figure 2L). Under more stringent conditions, sequential killing assays showed that CAR5 and CAR6 NK-92MI cells exhibited marked cytotoxicity reduction after two rounds, whereas CAR4 NK-92MI cells retained significantly stronger activity even after three rounds (Figure 2M). In the low antigen density model, despite only a one-third reduction in antigen density (Figure S5C), CAR4 NK-92MI cells exhibited superior cytotoxic activity compared to other constructs (Figure 2N).

Cytokine production (IFN-γ and TNF-α) is tightly regulated and requires a higher activation threshold than cytotoxicity 18. Intracellular flow cytometry showed that neither mock nor CAR NK-92MI cells produced IFN-γ or TNF-α at steady state or upon B16 stimulation. CAR4 NK-92MI cells produced the highest levels of both cytokines following B16-B7H3 stimulation (Figure 2O-P; Figure S5D). Collectively, these data demonstrated that CAR4 was the most potent among the 9 tested constructs in enhancing NK cell-mediated effector functions.

### Expression and function of CAR constructs in primary and iPSC-derived NK cells

**Figure 3:**
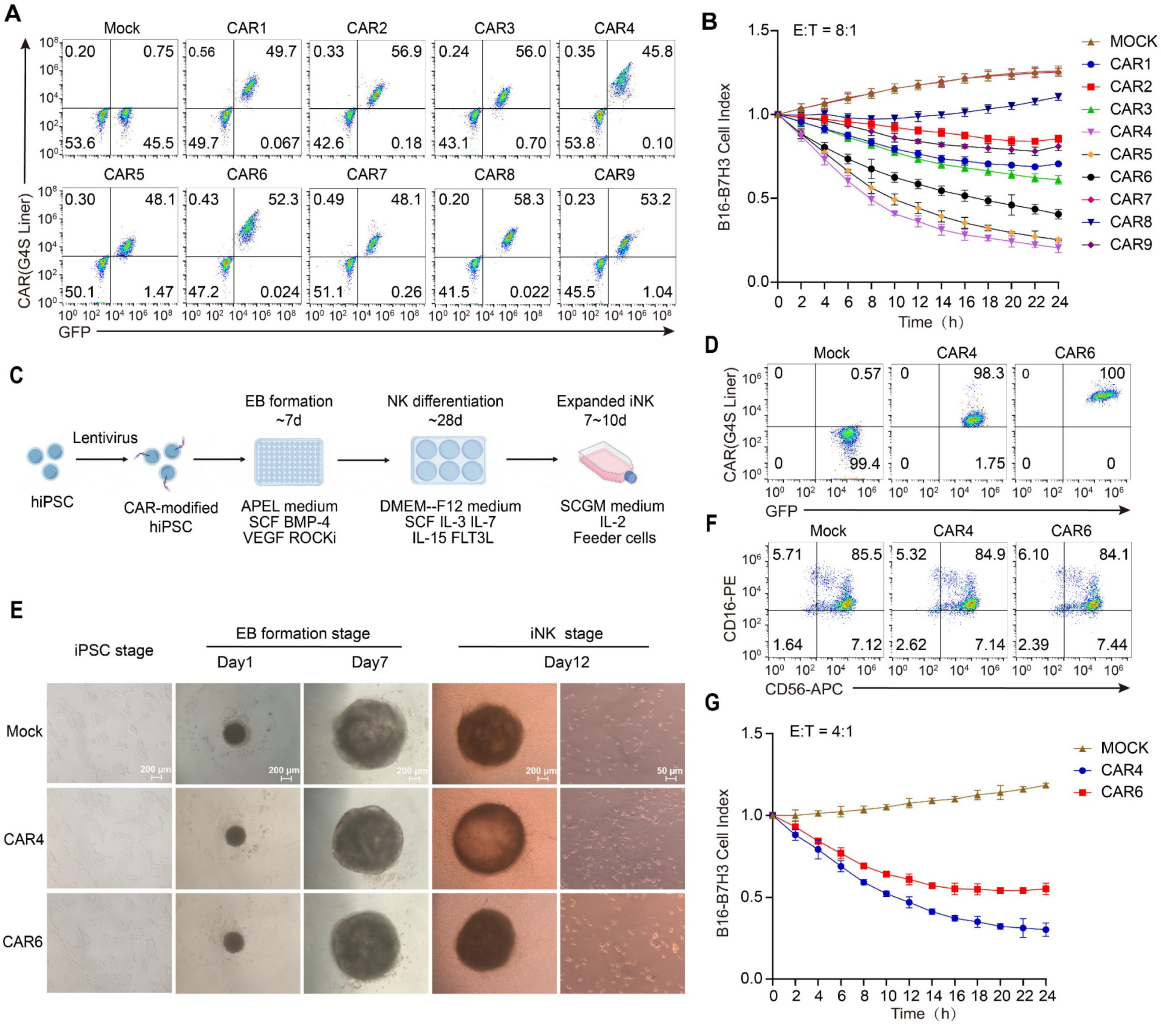
** Expression and function of NK-CARs in primary and iPSC-derived NK cells. (A)** Flow cytometry analysis of CAR expression in primary NK cells. **(B)** Primary NK cells in (**A**) were used as effectors at 8:1 ratio in Cellcyte X imaging system-based function assay with B16-B7H3 target cells over a 24h time-course. B16-B7H3 cells were transfected to express mCherry. Red fluorescence data were recorded and normalized to the initial scan data to calculate cell index. Values are represented as means ± SD, n = 4 technical replicates per group. **(C)** Scheme of CAR-NK differentiation from human iPSCs. IPSCs were engineered to express CAR molecules via lentiviral transfection, followed by puromycin selection to establish stable transfected CAR-iPSCs. A total of 10000 single-cell dissociated iPSCs were seeded in a U-bottom 96-well plate to form EBs. After day 7, EBs were then directly transferred into six-well plates with NK cell differentiation medium for 4 weeks. These NK cells can be used directly or after expansion with feeder cells. **(D)** Flow cytometry analysis of CAR expression in human iPSCs. **(E)** Left panel: morphology of CAR-modified iPSCs. Middle panel: morphology of EBs at day1 and day7. Scale bars represent 200 μm. Right panel: differentiated iNK cells 12 days post-NK differentiation. Scale bars represent 50 μm. **(F)** Flow cytometry analysis of CD16 and CD56 expression of iNK cells. **(G)** Cytolysis ability of iPSC-NK populations against B16-B7H3 target cells. Values are represented as means ± s.d. n = 4 technical replicates per group.

To establish the translational relevance for clinical applications, we further validated its functionality in primary NK cells and induced pluripotent stem cell (iPSC)-derived NK cells. Primary NK cells were isolated from peripheral blood, activated via magnetic beads, and subsequently engineered into CAR NK cells using electroporation. Transduction efficiencies were consistent across all constructs, averaging approximately 50% (Figure 3A). Continuous killing assays yielded results consistent with those observed in NK-92MI cells: CAR4, CAR5 NK cells exhibited notable sustained cytotoxicity, with CAR4 NK cells displaying the most potent and prolonged cytolytic activity (Figure 3B).

IPSC-derived CAR NK cells were generated using a standardized and efficient “spin embryoid body (EB)” differentiation protocol (Figure 3C). To rigorously compare T cell-derived signaling motifs (CD28-CD3ζ) with NK-optimized motifs (NKG2DTM-2B4-FCER1G), CAR4 and CAR6 were expressed in human iPSCs (Figure 3D). These iPSCs maintained robust stemness, as evidenced by the expression of SSEA and TRA-1-81 markers (Figure S6). Notably, in contrast to the expression profile in NK-92MI cells, CAR4 expression in iPSCs was significantly lower than that of CAR6 (Figure 3D). By day 12 of NK cell differentiation, a substantial population of suspended cells had emerged (Figure 3E). Flow cytometric analysis confirmed that over 90% of these suspended cells were NK cells, with the majority being CD16-positive mature NK cells (Figure 3F). Functional assessments of CAR-expressing iPSC-derived NK cells revealed that, despite the lower expression level of CAR4, longitudinal *in vitro* cytotoxicity assays consistently demonstrated superior activity of CAR4 iPSC-NK cells compared to CAR6 iPSC-NK cells (Figure 3G). These findings not only validate the robustness of the CAR design across diverse NK cell platforms but also underscore its broad clinical translatability.

### The CAR construct incorporating NKG2DTM-2B4-FCER1G is also effective when targeting CD19

**Figure 4:**
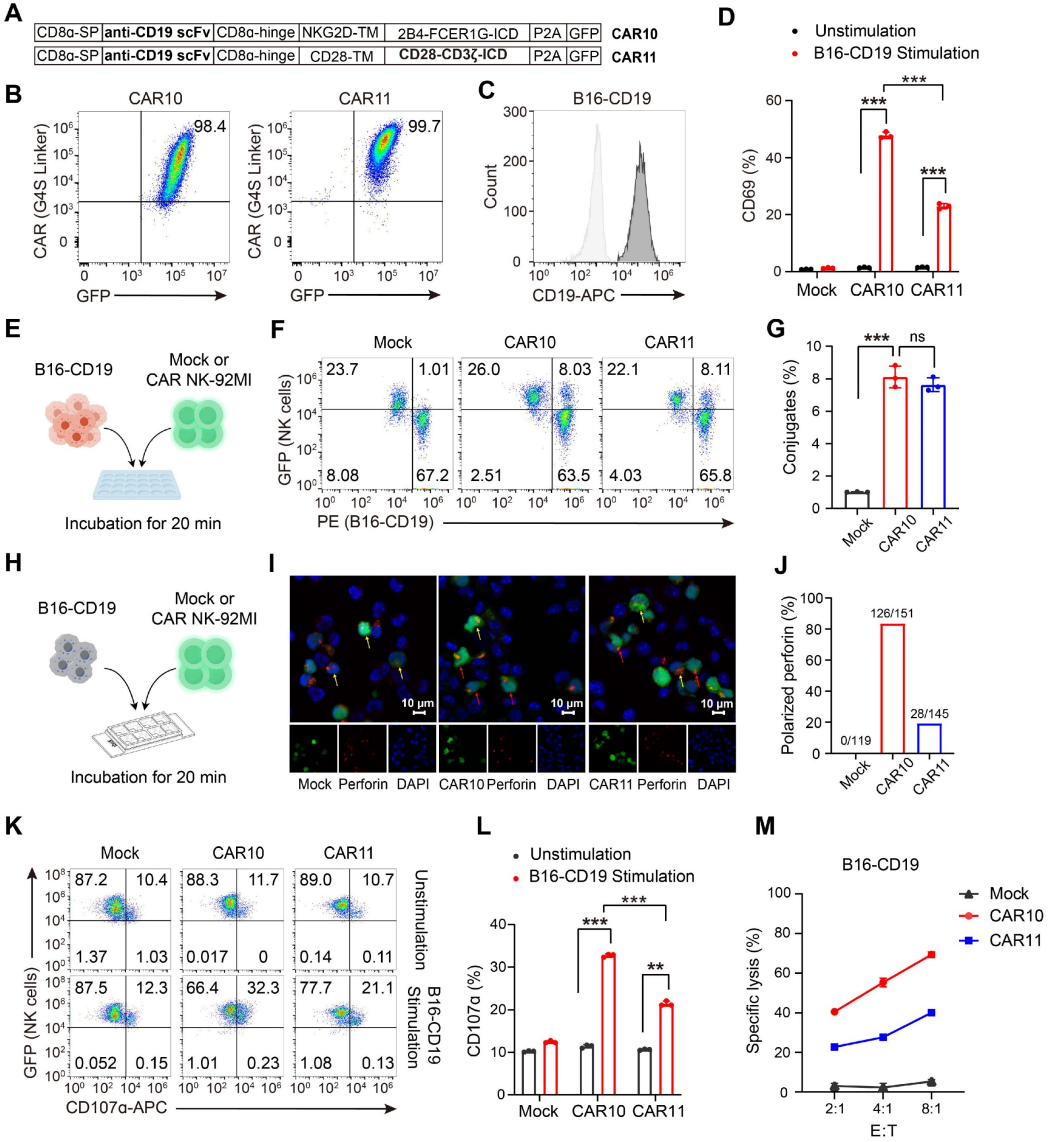
** The NKG2DTM-2B4-FCER1G CAR construct retains efficacy when targeting CD19.** (**A**) Schematic illustration of the NK cell-specific CAR constructs targeting CD19. (**B**) Expression of GFP and CAR molecule in NK-92MI cells assessed by flow cytometry. (**C**) Construction of B16 cell line overexpressing CD19 by lentiviral transfection. (**D**) Summary of CD69 expression in response to B16-CD19 cells (n = 3). (**E**) Schematic illustration of *in vitro* conjugate formation assays. B16-CD19 cells labeled with Orange Fluorescence (PE channel) were incubated with GFP-expressing Mock, CAR10 and CAR11 NK cells for 20 min. (**F**) Representative conjugate formation**.** NK cell-target cell conjugates are identified as PE^+^GFP^+^ cells in the upper right quadrant. (**G**) Summary of conjugate formation (n = 3). (**H**) Schematic of *in vitro* granule polarization assays. (**I**) Representative confocal microscopy images of GFP-expressing Mock, CAR10 and CAR11 NK-92MI cells cocultured with B16-B7H3 cells and stained for Alexa 594-conjugated perforin mAb with a 60× oil immersion objective. Perforin-containing granules were considered polarized when most of the randomly distributed fluorescence was concentrated in the quadrant of the NK-92MI cell. Red and yellow arrow indicate polarized and non-polarized granules respectively. (**J**) Summary of granule polarization, more than 100 NK cells per group were scored using a 40× objective to calculate the percentage of polarization. (**K**) Representative degranulation assayed by CD107α of Mock, CAR10 and CAR11 NK-92MI cells against B16-CD19 target cells. (**L**) Summary of degranulation (n = 3). (**M**) Representative specific killing of B16-CD19 cells at different cell-to-cell ratios (n = 3). Data are represented as mean ± s.d. Two-way ANOVA followed by Sidak's (**D, G, L**) multiple comparisons test was used.

To expand its potential application in hematological malignancies, we next evaluated whether the NKG2DTM-2B4-FCER1G CAR construct retains efficacy when redirected to target CD19. A CD19-targeting CAR (designated CAR10) was generated using an anti-CD19 scFv (Figure 4A-B). CAR11, incorporating CD28 and CD3ζ, served as a control. A B16 cell line overexpressing CD19 (B16-CD19) was constructed by lentiviral transfection and verified by flow cytometry analysis (Figure 4C). Compared to CAR11, CAR10 significantly promoted NK cell activation as indicated by increased CD69 expression (Figure 4D). Both CAR10 and CAR11 NK-92MI efficiently formed conjugates with B16-CD19 cells (Figure 4E-G). In terms of granule polarization, CAR10 was clearly superior to CAR11 (Figure 4H-J), and achieved a corresponding increase in degranulation (Figure 4K-L) and cytotoxicity (Figure 4M) against B16-CD19 cells. Collectively, these findings confirmed that the NKG2DTM-2B4-FCER1G CAR construct retained efficacy when targeting CD19.

### The NKG2DTM-2B4-FCER1G CAR construct activates a majority of CD16-downstream signaling pathways

We next investigated the signaling cascades triggered by the NKG2DTM-2B4-FCER1G CAR upon target engagement. Considering that CD16 preferentially uses the FCER1G-encoded γ chain for signal transduction 16, with 2B4 known to enhance CD16-dependent degranulation and cytokine production 18, 19, we sought to determine whether this CAR recapitulates CD16-mediated signaling. The downstream signaling events of CD16 are well characterized, including SYK and ZAP70 17, 26, PI3K 27, 28, (GDP)-guanosine triphosphate (GTP) exchange factor (VAV1) 29, 30, ERK 25, 31, 32, PLC-γ 33.

**Figure 5:**
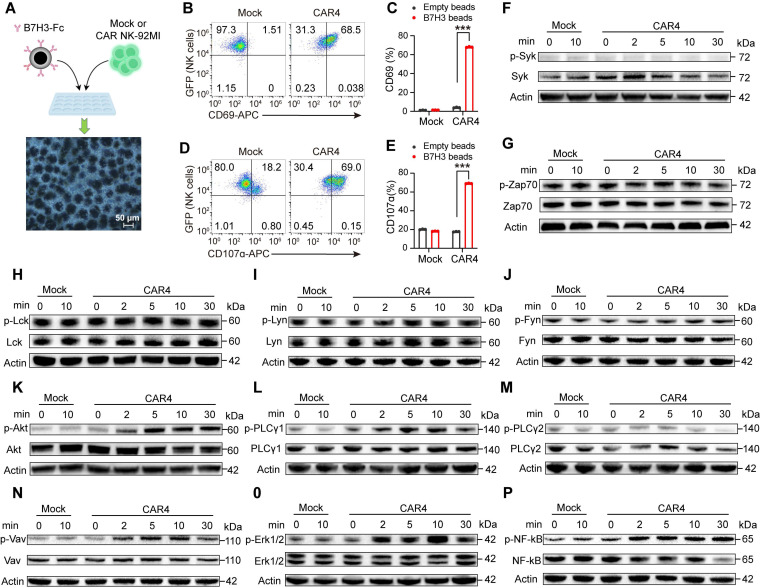
** The NKG2DTM-2B4-FCER1G CAR construct mediates activation of the AKT, PLCγ1, VAV1, ERK, and NF-κB pathways. (A)** Schematic representation of NK cell activation using protein A-coupled beads coated with recombinant B7H3-Fc chimera. Following incubation, beads were removed with a magnet, and purified NK cells were subjected to flow cytometry and immunoblot analysis. **(B)** Representative flow cytometry plots showing CD69 expression after stimulation with B7H3-Fc-coated beads. **(C)** Summary of CD69 expression (n = 3). **(D)** Representative flow cytometry plots of degranulation (assessed by CD107α expression) in Mock and CAR4 NK-92MI cells after stimulation with B7H3-Fc-coated beads.** (E)** Summary of degranulation (n = 3). **(F-P)** Mock and CAR4 NK-92MI cells were stimulated with B7H3-Fc-coated beads for the indicated times (0 min = unstimulated). Whole-cell lysates were analyzed by Western blotting for: phospho-SYK (Tyr525/526) and total SYK (n = 2) (**F**). phospho-ZAP70 (Tyr319) and total ZAP70 (n = 2) (**G**). phospho-LCK (Tyr394) and total LCK (n = 2) (**H**). phospho-LYN (Tyr397) and total LYN (n = 2) (**I**). phospho-FYN (Tyr530) and total FYN (n = 2) (**J**). phospho-AKT1 (S473) + AKT2 (S474) + AKT3 (S472) and total AKT1 + AKT2 + AKT3 (n = 4) (**K**). phospho-PLCγ1 (Tyr783) and total PLCγ1 (n = 3) (**L**). phospho-PLCγ2 (Tyr1217) and total PLCγ2 (n = 5) (**M**). phospho-VAV1 (Tyr174) and total VAV1 (n = 6) (**N**). phospho-ERK1/2 (Thr202/Tyr204) and total ERK1/2 (n = 3) (**O**). phospho-NF-κB p65 (Ser536) and total NF-κB p65 (n = 3) (**P**). Data are represented as mean ± s.d. Two-way ANOVA followed by Sidak's (**C**, **E**) multiple comparisons test was used.

To rule out potential interference from B16 cells, CAR4 NK-92MI cells were stimulated with either uncoated beads or beads conjugated with recombinant human B7H3-Fc chimera (Figure 5A). B7H3-Fc-coated beads specifically induced CD69 upregulation and degranulation in CAR4 NK-92MI cells, but not in mock cells (Figure 5B-E), validating this model for signaling pathway analysis.

NK cell activation involves the coupling of proximal signals including SYK and SRC family protein tyrosine kinases to distal downstream effectors 34. We first examined proximal signaling events. Phosphorylation of ZAP70, and three SRC family kinases was detected in both mock and CAR4 NK-92MI cells under basal conditions, with no further enhancement upon stimulation with B7H3-Fc-coated beads (Figure 5F-J). We then analyzed downstream signaling cascades. Upon stimulation with B7H3-Fc-conjugated beads, CAR4 specifically induced increased tyrosine phosphorylation of AKT (Figure 5K), PLCγ1 (Figure 5L-M), VAV1 (Figure 5N), and ERK1/2 (Figure 5O). Consistent with its ability to promote cytokine production, the CAR also activated the NF-κB pathway (Figure 5P). These results demonstrated that the NKG2DTM-2B4-FCER1G CAR activated a majority of CD16-associated signaling pathways.

### Anti-tumor activity of NKG2DTM-2B4-FCER1G CAR-expressing NK-92MI cells in diverse tumor models

**Figure 6:**
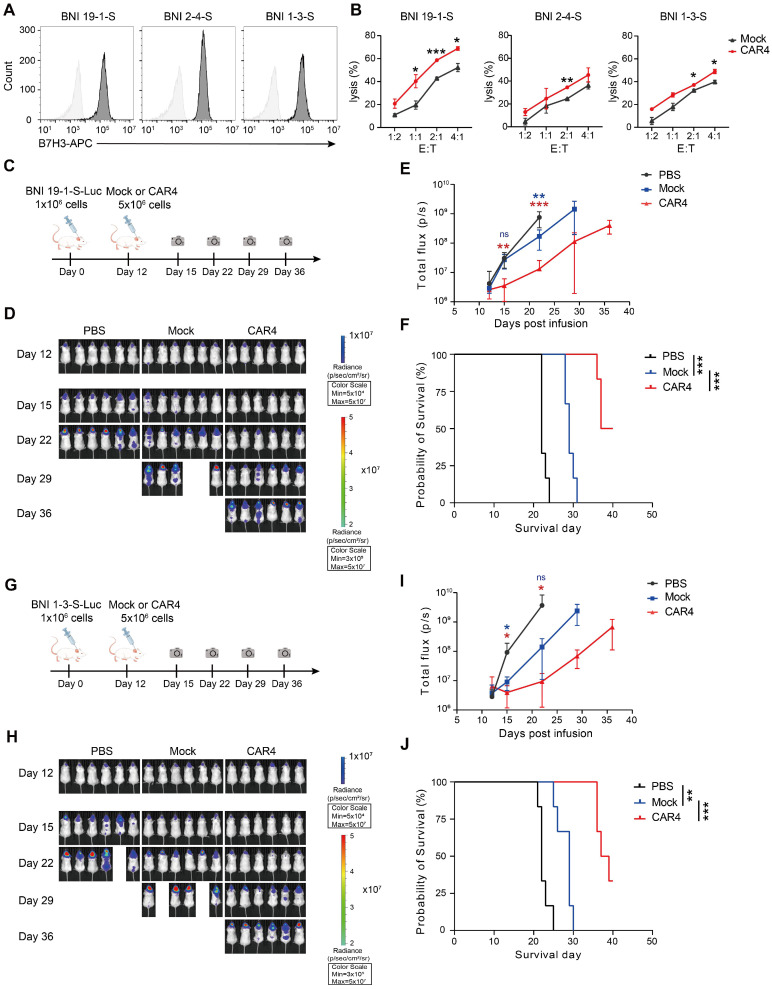
** Anti-tumor activity of CAR4 NK-92MI cells against glioma stem cells. (A)** Surface expression of B7H3 in glioma stem cell lines (BNI-19-1-S, BNI-2-4-S, BNI-1-3-S) as assessed by flow cytometry. **(B)** Representative cytotoxicity of Mock and CAR4 NK-92MI cells against BNI-19-1-S, BNI-2-4-S, and BNI-1-3-S cells at various effector-to-target ratios (n = 4). **(C)** Schematic of the *in vivo* study: NPG mouse xenografts were established with luciferase (luc)-expressing BNI-19-1-S cells and treated with Mock or CAR4 NK-92MI cells. **(D)** Tumor burden monitored by bioluminescent imaging (BLI). **(E)** Quantification of tumor burden based on BLI total flux (photons/sec). Data are presented as mean ± s.d. **(F)** Kaplan-Meier survival curves for experimental groups. Significance was determined by log-rank test (n = 6 per group). Two independent experiments were performed.** (G)** Schematic of the *in vivo* study: NPG mouse xenografts were established with luc-expressing BNI-1-3-S cells and treated with locoregionally administered Mock or CAR4 NK cells. **(H)** Tumor burden monitored by BLI. **(I)** Quantification of tumor burden based on BLI total flux (photons/sec). Data are presented as mean ± s.d. **(J)** Kaplan-Meier survival curves for experimental groups. Significance was determined by log-rank test (n = 6 per group). Two independent experiments were performed. Statistical analyses were performed using one-way ANOVA (**E**, **I**) or two-way ANOVA (**B**) followed by Sidak's multiple comparisons test.

We further evaluated the anti-tumor activity of the CAR-expressing NK-92MI cells in both solid and hematologic tumor models. For solid tumor testing, three glioma stem cell lines were confirmed to express high levels of B7H3 (Figure 6A). CAR4 NK-92MI cells exhibited superior cytolytic activity against these lines compared to mock NK-92MI cells (Figure 6B). In an orthotopic model using the BNI-19-1-S glioma stem cell line (Figure 6C), CAR4 NK-92MI cell treatment resulted in significant tumor growth suppression by day 15 (88% reduction in tumor burden, as reflected by bioluminescent flux), sustained inhibitory effects through day 35, and a prolonged survival advantage (median survival: 37 days vs. 22 days in controls) (Figure 6D-F). Similar results were observed in the BNI-1-3-S orthotopic glioma model (Figure 6G), where CAR4 NK-92MI cells conferred enhanced anti-tumor efficacy and survival (median survival: 36 days vs. 21 days in controls) (Figure 6H-J).

**Figure 7:**
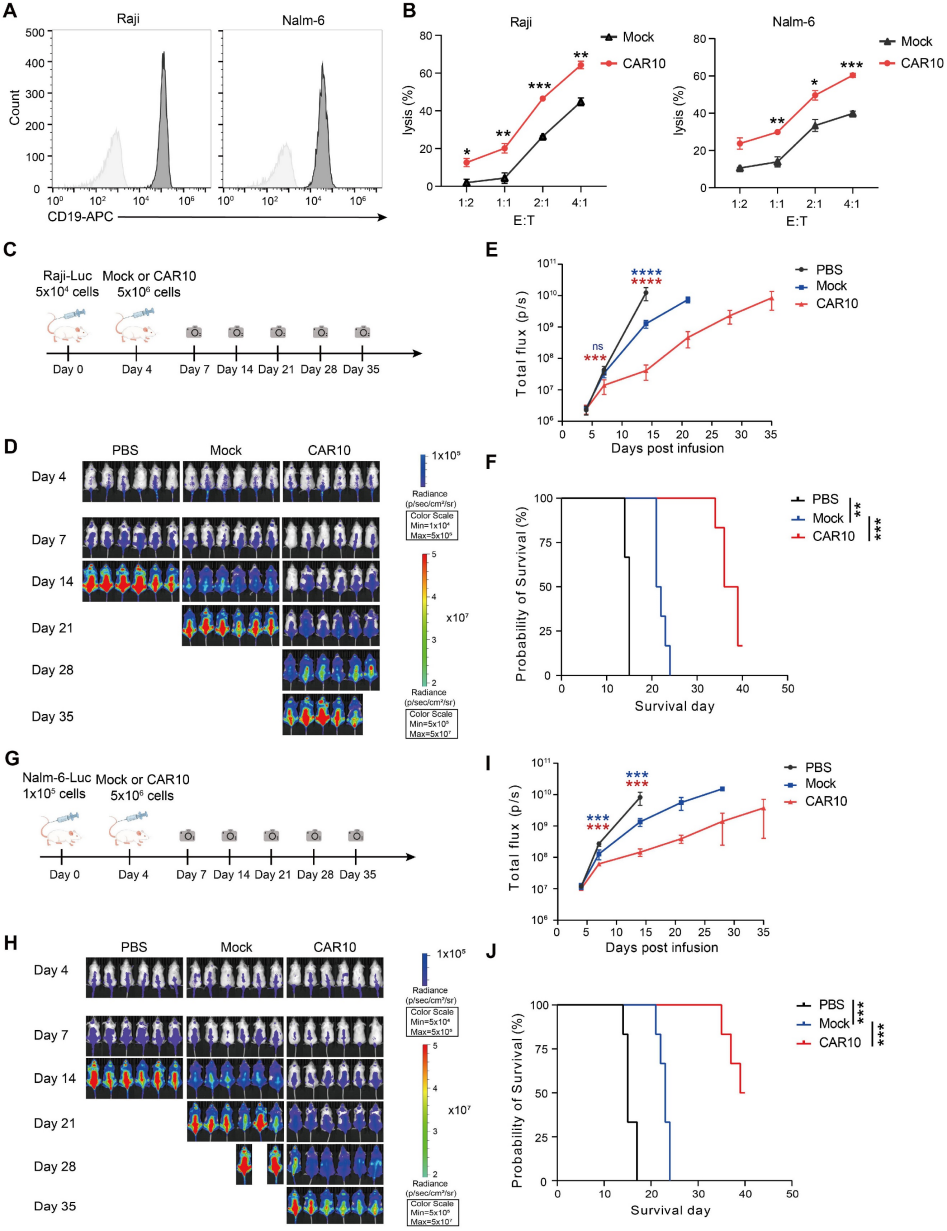
** Anti-tumor activity of CAR10 NK-92MI cells against B-cell lymphoma. (A)** Surface expression of CD19 in Raji and Nalm-6 cells as assessed by flow cytometry. **(B)** Representative cytotoxicity of Mock and CAR10 NK-92MI cells against Raji and Nalm-6 cells at various effector-to-target ratios (n = 4). **(C)** Schematic of the *in vivo* study: NPG mouse xenografts were established with luciferase (luc)-expressing Raji cells and treated with Mock or CAR10 NK-92MI cells. **(D)** Tumor burden monitored by bioluminescent imaging (BLI). **(E)** Quantification of tumor burden based on BLI total flux (photons/sec). Data are presented as mean ± s.d. **(F)** Kaplan-Meier survival curves for experimental groups. Significance was determined by log-rank test (n = 6 per group). Two independent experiments were performed.** (G)** Schematic of the *in vivo* study: NPG mouse xenografts were established with luc-expressing Nalm-6 cells and treated with Mock or CAR10 NK-92MI cells. **(H)** Tumor burden monitored by BLI. **(I)** Quantification of tumor burden based on BLI total flux (photons/sec). Data are presented as mean ± s.d. **(J)** Kaplan-Meier survival curves for experimental groups. Significance was determined by log-rank test (n = 6 per group). Two independent experiments were performed. Statistical analyses were performed using one-way ANOVA (**E**, **I**) or two-way ANOVA (**B**) followed by Sidak's multiple comparisons test.

For hematologic malignancy testing, we assessed CAR10 NK-92MI cells in lymphoma models. Raji and Nalm-6 cells were confirmed to express high levels of CD19 (Figure 7A), and CAR10 NK-92MI cells displayed superior *in vitro* cytotoxicity against these lines compared to mock cells (Figure 7B). In a Raji lymphoma mouse model (Figure 7C), a single intravenous infusion of CAR10 NK-92MI cells resulted in significantly anti-tumor activity and prolonged survival (median survival: 36 days vs. 15 days in controls) (Figure 7D-F). Similarly, in a Nalm-6 lymphoma model (Figure 7G), CAR10 NK-92MI cells achieved better tumor control and a superior survival benefit (median survival: 39 days vs. 15 days in controls) (Figure 7H-J).

To elucidate the *in vivo* distribution and pharmacokinetics of intravenously administered CAR NK cells, we re-established the Raji murine model. The CAR NK cells were engineered to express green fluorescent protein (GFP) during construction. We therefore utilized the IVIS Spectrum *In vivo* Imaging System to directly capture fluorescent signals for assessing cellular distribution. Results showed that at 2 h post-injection, Mock and CAR10 NK-92MI cells were predominantly distributed around the injection site and in the lungs (Figure S7A). By 24h, the infiltration range of CAR10 NK-92MI cells had expanded significantly compared with the mock group. On day 3 post-injection, a substantial number of CAR10 NK-92MI cells remained detectable, whereas NK cells were nearly undetectable in the mock group. Flow cytometry data also revealed that CAR10 NK-92MI cells exhibited a higher proportion than the mock group at 24 h and 48 h post-injection (Figure S7B). These findings demonstrated that CAR NK cells could persist *in vivo* for at least 72 h, with activated CAR NK cells exhibiting enhanced *in vivo* persistence.

## Discussion

The two-signal activation theory in T cells, wherein the first signal is delivered by antigen peptide-MHC complexes and the second by costimulatory molecules such as CD28, has directly shaped the classical CAR T design (CD28/4-1BB-CD3ζ). This underscores the principle that CAR architectures should be tailored to the intrinsic activation mechanisms of specific immune cell lineages. Guided by the synergistic interactions between NK cell activating receptors, we designed a panel of CAR constructs. Given that a prior study highlighted the critical role of the NKG2D transmembrane domain in mediating robust antigen-specific signaling in CAR NK cells 10, we generated distinct CAR variants incorporating this domain, paired with either 2B4 or DAP10 as co-activation domains, and FCER1G or CD3ζ as signaling domains. NK cell cytotoxicity is primarily exerted via the granule exocytosis pathway, which proceeds through discrete steps: (i) conjugate formation with target cells, (ii) polarization of cytotoxic granules toward the target, and (iii) degranulation (exocytosis of secretory granules) 12, 35, 36. Our functional assays demonstrated that among the 9 constructs, the CAR incorporating NKG2D-2B4-FCER1G was the most potent in enhancing NK cell-mediated effector functions. Furthermore, validation in primary NK cells and induced pluripotent stem cell (iPSC)-derived NK cells confirmed the translational relevance of this CAR design for clinical applications.

The evaluation of CAR-mediated signaling pathways is often based on the corresponding receptors. For instance, NKG2D associates with the transmembrane adaptor DAP10 for intracellular signaling, and chimeric receptors containing the DAP10 intracellular domain can largely recapitulate NKG2D-mediated activation pathways 17, 37, 38. Using a bead-based stimulation model, we confirmed that the NKG2D-2B4-FCER1G CAR activated most of the signaling pathways downstream of CD16.

Despite CD28 being traditionally regarded as a T cell-centric co-stimulatory molecule, CAR6, incorporating the CD28 co-activation domain and CD3ζ signaling domain, also significantly enhanced NK-92MI cell activity. This is consistent with reports that CD28 inclusion in CAR constructs can promote CAR NK cell persistence and sustained cytotoxicity by recruiting key kinases such as LCK and ZAP70 11. Additionally, CD28 expression has been detected in peripheral blood NK cells from healthy adults (albeit antibody-dependent) 39. the NK leukemia cell line YT2C2 expresses CD28 and exhibits preferential cytotoxicity against B7-bearing B lymphoblasts 40. Our observation that NK-92MI cells express high levels of CD28 further supports its potential role in NK cell cytotoxicity.

Collectively, our findings demonstrate that the NKG2DTM-2B4-FCER1G CAR construct potently enhances NK cell functionality by activating phosphorylation-dependent signaling through AKT, VAV1-ERK, PLCγ1, and NF-κB pathways. These activation events are sufficient to promote granule polarization, degranulation, cytotoxicity, cytokine production, and *in vivo* anti-tumor activity. This optimized CAR design thus holds promise for advancing NK cell-based immunotherapies.

## Materials and Methods

### Cell lines

NK-92MI cells were purchased from Procell Life Science & Technology Co., Ltd (Wuhan, China) and cultured in NK-92MI cell culture medium (MEMα supplemented with 0.2 mM inositol, 0.1 mM β-mercaptoethanol, 0.02 mM folic acid, 12.5% HS, 12.5% FBS, 1% P/S). Three glioma stem cell lines (BNI-19-1-S, BNI-2-4-S, BNI-1-3-S) were previously established in our laboratory. Briefly, glioblastoma tissue samples (IDH-wildtype, CNS grade 4, according to the 2021 WHO Classification of CNS Tumors) were obtained from patients who underwent surgical resection at Beijing Tiantan Hospital. Tissues were washed with pre-cooled DMEM, minced gently, and digested with 10 mL trypsin at 37 °C for 20 min. The cell suspension was filtered through a 200-mesh sieve to obtain single cells, which were cultured in serum-free medium (DMEM/F12 supplemented with 2% B27, 20 ng/mL EGF, and 20 ng/mL FGF). The Raji and Nalm-6 human B-cell lymphoma cell lines, and the murine B16F10 melanoma cell line (hereafter referred to as B16) were obtained from the Cell Resource Center, Institute of Basic Medical Sciences (CAMS/PUMC, Beijing, China) and cultured in RPMI-1640 supplemented with 10% FBS and 1% P/S. Except for NK-92MI cells, all other cell lines were transduced with lentivirus encoding firefly luciferase or mCherry to facilitate cytotoxicity assays.

### Generation of CAR NK cells

CAR constructs consisted of: a CD8A leader sequence (GenBank: NM_001145873.1), a CD8A hinge region, a transmembrane domain (NKG2D [GenBank: AF461811.1] or CD28 [GenBank: AF222341.1]), co-activation domains (2B4 [GenBank: AF107761.2], DAP10 [GenBank: AF172929.2], or CD28), and signaling domains (CD3ζ [GenBank: NM_198053.3] or FCER1G [GenBank: NM_004106.2]). The single-chain variable fragment (scFv) of an anti-B7H3 antibody (US20100143245A1) or the anti-CD19 scFv from the FMC63 monoclonal antibody was inserted between the leader sequence and hinge region. Since NKG2D is a type II integral membrane protein, the amino acid sequence of its transmembrane domain was inverted to maintain the natural N-terminal-to-C-terminal orientation. All CAR constructs were synthesized by Azenta Life Sciences and cloned into the pCMV-PB-puro plasmid (Celetrix Technologies).

Primary NK cells were isolated from peripheral blood and cultured in SCGM medium (CellGenix, 20802-0500) supplemented with 200 U/mL hIL-2, activated via magnetic beads (Miltenyi Biotec, 130-094-483). After collecting and washing NK cells with PBS, 5 × 10^6^ -1×10^7^ cells were electroporated with 8 μg screened plasmids using the Celetrix electroporator (Model CTX-1500A LE+) at 430 V with a single pulse of 30 ms. Electroporated NK cells were cultured in 6-well plates. After 72 h, NK-92MI cells were subjected to selection with 2 μg/mL puromycin (Solarbio) for a minimum of 2 weeks. The expression of CAR molecules was detected as both positive for GFP and anti-G4S linker (B02H1) antibody (Hycells, GS-ARNC25).

### NK cell derivation from CAR-expressing iPSCs

Human induced pluripotent stem cells (iPSCs) were a gift from Zhiyi Liao. iPSCs were cultured in mTeSR™ Plus medium (STEMCELL, 100-0276). To generate CAR-expressing iPSCs, cells were transduced with lentivirus encoding CAR constructs and selected with puromycin to establish stable lines. For embryoid body (EB) formation, 10000 single iPSCs were seeded in U-bottom 96-well plates in APEL medium (STEMCELL, 05270) supplemented with 40 ng/mL SCF (PeproTech, 300-07-10), 20 ng/mL VEGF (R&D Systems, BT-VEGF-020), and 20 ng/mL BMP-4 (R&D Systems, 314-BP-010/CF). On day 7, EBs were transferred to 6-well plates containing DMEM/F-12 medium (STEMCELL, 10565018) supplemented with 5 ng/mL IL-3 (PeproTech, 200-03-2UG; first week only), 10 ng/mL IL-15 (PeproTech, 200-15-10UG), 20 ng/mL IL-7 (PeproTech, 200-07-10UG), 20 ng/mL SCF, and 10 ng/mL FLT3L (PeproTech, 300-19-2ug) for 4 weeks, with half-medium changes weekly. NK cells were expanded using irradiated mbIL-21-expressing artificial antigen-presenting cells (aAPCs) in SCGM medium (CellGenix, 20802-0500) supplemented with 200 U/mL hIL-2.

### Genetic engineering of B16 cells

Human B7H3 (NM_001024736) and CD19 (NM_001178098.2) sequences were cloned into the lentiviral vector pHS-B-0085. Lentiviral particles were generated by transfecting Lenti-X 293T cells (Takara) with 6.25 μg of the expression vector, 13 μg pCMV-dR8.9-dvpr, and 6.5 μg pCMV-VSV-G (Beijing Syngenbio Co., Ltd.) using polyethylenimine per 15 cm dish. Particles were harvested, concentrated using a Lentiviral Concentration Kit (Beijing Syngenbio Co., Ltd.), and used to transduce B16 cells at a multiplicity of infection (MOI) of 20. Transduced cells were selected with 2 μg/mL puromycin for at least 2 weeks. B7H3 and CD19 expression was confirmed by flow cytometry using anti-human B7H3 (BioLegend, Cat# 351005) and anti-human CD19 (BioLegend, Cat# 302211) antibodies, respectively.

### Cell cycle analysis

Cell cycle distribution was analyzed using the Cell Cycle Analysis Kit (Beyotime Biotechnology, C1052). Log-phase NK-92MI cell lines were harvested, fixed in 70% ethanol at 4 °C for 6 h, and stained with propidium iodide (0.05 mg/mL) and RNase A (1 mg/mL) in the dark for 30 min. Samples were analyzed by flow cytometry, and the proportions of cells in G1, S, and G2/M phases were determined based on DNA content (propidium iodide fluorescence intensity).

### Cell proliferation assay

NK-92MI cell lines (1 × 10³ per well) were seeded in 96-well plates and cultured for 72 h. CCK-8 solution (Beyotime Biotechnology, C0038) was added for 2 h, and absorbance at 450 nm was measured.

### Apoptosis assays

Log-phase NK-92MI cell lines (1 × 10⁵) were harvested and stained with Annexin V (Beyotime Biotechnology, C1065M) in the dark at room temperature for 15 min. Apoptosis was analyzed by flow cytometry.

### Conjugate formation assays of NK-92MI cells against targets

B16, B16-B7H3, and B16-CD19 cells were labeled with the Cell Explorer™ Live Cell Labeling Kit *Orange Fluorescence* (AAT Bioquest, Cat# 22622; PE channel) at 37 °C for 15 min. Mock or CAR NK-92MI cells (5 × 10⁵) were co-incubated with target cells (1 × 10⁶) in 1 mL of NK-92MI medium at 37 °C for 20 min, then fixed with 4% paraformaldehyde (PFA; Biosharp). Conjugates (GFP⁺ NK cells and PE⁺ targets) were detected by flow cytometry.

### Granule polarization assay

μ-Slide 8-well plates (ibidi, Cat# 80826) were coated with Poly-L-Lysine (Sigma-Aldrich, Cat# 25988-63-0) at 37 °C for 12 h. B16-B7H3 or B16-CD19 cells (1.5 × 10⁵) in 300 μL RPMI 1640 complete medium were added to each well; after adhesion, medium was aspirated, and 1.5 × 10⁵ mock or CAR NK-92MI cells in 300 μL NK-92MI medium were added and incubated at 37 °C for 20 min. This cell density (3 × 10⁵ cells per 1.0 cm²) ensured full effector-target contact. Cells were fixed with 4% PFA for 10 min, permeabilized with 0.1% Triton X-100 (Sigma-Aldrich, Cat# SLBS6421) for 10 min, and blocked with goat serum (ZSGB-BIO, Cat# ZLI-9022) for 30 min at room temperature. Cells were stained with anti-perforin antibody (10 μg/mL; BioLegend, Cat# 308124) for 1 h at room temperature, and nuclei were counterstained with DAPI. Images were acquired using a confocal microscope (ZEISS LSM 880) with a 40× or 60× oil immersion objective. At least 100 NK cells per group were evaluated under 40× magnification to determine the percentage of granule polarization.

### Degranulation Assays

Mock or CAR NK-92MI cells (5 × 10⁵) were co-incubated with target cells (1 × 10⁶) in 1 mL of NK-92MI medium at 37 °C for 2 h. Cells were fixed with 4% PFA, washed, and stained with anti-CD107a antibody (BioLegend, Cat# 328620) at 4 °C for 30 min. Degranulation was analyzed by flow cytometry.

### Cytotoxicity assays

Cytotoxicity was evaluated using luciferase-expressing target cells. Mock or CAR-NK-92MI cells were co-cultured with target cells (1 × 10⁴) in 100 μL of NK-92MI medium at varying effector-to-target (E : T) ratios for 4 h. Bright-Lite® Luciferase Assay System substrate (Vazyme, Cat# DD1204-02; 100 μL) was added, and luminescence was measured using a plate reader. For longitudinal assays, B16-B7H3 cells were transduced with mCherry and selected with blasticidin to generate stable lines. As for continuous killing assays, NK cells were used as effectors at indicated ratios in with B16-B7H3 target cells using the Cellcyte X imaging system over a 24 h time-course. All red fluorescence data were recorded and normalized to the initial scan data to calculate cell index. For sequential killing assays, an equivalent count of target cells was added to the mixtures at an interval of 8 h.

### Fc fusion protein and bead stimulation

Recombinant human B7H3-Fc fusion protein was purchased from BioLegend (Cat# 564606). Protein A-conjugated 50-μm beads (GenScript, Cat# L00273) were washed twice with PBS (1 × 10⁵ beads/200 μL slurry), coated with 4 μg fusion protein in 200 μL PBS, and rotated at room temperature for 1 h. Beads were washed twice with PBS and resuspended in 200 μL NK-92MI medium. Mock or CAR NK-92MI cells (1 × 10⁶) were incubated with 100 μL of fusion protein-coated or uncoated beads in 1 mL of NK-92MI medium at 37 °C for 2 or 6 h. Beads were removed with a magnet, and cells were harvested for flow cytometry or immunoblotting.

### Flow cytometry

For extracellular staining, cells were harvested, washed with PBS, and stained at 4 °C for 30 min in the dark. For intracellular staining, cells were fixed with 4% PFA for 10 min, permeabilized with 0.1% Triton X-100 for 10 min, and stained at 4 °C for 30 min in the dark. Samples were analyzed using an Accuri C6 Plus flow cytometer (BD) and FlowJo software v.10.8.1.

### Western blotting

Mock or CAR-NK-92MI cells (4 × 10⁶) were harvested after bead stimulation, washed with ice-cold PBS, and lysed in pre-chilled RIPA buffer (Solarbio) supplemented with 1% proteinase and phosphatase inhibitor cocktails (Selleck). Lysates (15 μL) were separated by 4%-20% SDS-PAGE (GenScript, Cat# M00656) and transferred to PVDF membranes. Membranes were blocked with 5% nonfat milk or BSA in PBST for 1 h at room temperature, then incubated overnight at 4 °C with primary antibodies: SYK (Tyr525/526; Cell Signaling Technology, Cat# 2710), SYK (Cell Signaling Technology, Cat# 2712), ZAP70 (Tyr319; Cell Signaling Technology, Cat# 2701), ZAP70 (Cell Signaling Technology, Cat# 2705), LYN (phospho Y397; Abcam, Cat# ab300118), LYN (Abcam, Cat# ab318191), LCK (phospho Y394; Abcam, Cat# ab318960), LCK (Immunoway, Cat# YT2546), FYN (phospho Y530; Abcam, Cat# ab188319), FYN (Abcam, Cat# ab125016), AKT1 (pS473) + AKT2 (pS474) + AKT3 (pS472; Abcam, Cat# ab192623), AKT1 + AKT2 + AKT3 (Abcam, Cat# ab185633), PLCγ1 (Tyr783; Cell Signaling Technology, Cat# 2821), PLCγ1 (Cell Signaling Technology, Cat# 5690), PLCγ2 (Tyr1217; Cell Signaling Technology, Cat# 3871), PLCγ2 (Cell Signaling Technology, Cat# 3872), VAV1 (phospho Y174; Abcam, Cat# ab76225), VAV1 (Immunoway, Cat# YT4862), ERK1/2 (Thr202/Tyr204; Cell Signaling Technology, Cat# 4370), ERK1/2 (Cell Signaling Technology, Cat# 4695), NF-κB p65 (Ser536; Cell Signaling Technology, Cat# 3033), and NF-κB p65 (Cell Signaling Technology, Cat# 8242). After three 10 min washes with PBST, membranes were incubated with secondary antibodies for 1 h at room temperature, and signals were detected using an ECL kit (Boster).

### *In vivo* tumor modelling

All mice were housed under specific pathogen-free (SPF) conditions in a barrier facility at the Beijing Neurosurgical Institute. Anti-tumor activity was evaluated in 5-6 weeks NOD.Cg-Prkdc^scid^IL2rg^tm1Vst^/Vst mice (Vitalstar Biotechnology Co., Ltd). Female mice were housed 3-5 per cage under a 12 h light/dark cycle (20-24 °C, 40-60% humidity). Tumor growth was monitored by bioluminescence using an IVIS Spectrum *In vivo* Imaging System (PerkinElmer) and quantified as total flux (photons/sec) using Living Image software (v4.4; PerkinElmer).

For orthotopic glioma models, female NPG mice were anesthetized with 3% isoflurane (induction) and 2% isoflurane (maintenance). A burr hole was drilled 2 mm lateral and 1 mm anterior to the bregma; a blunt needle (Hamilton Company) was inserted 3.5 mm deep, and 8 μL of BNI-19-1-S or BNI-1-3-S luciferase⁺ cells (1 × 10⁶) in PBS was injected over 2 min (needle retained for 1 min). After confirming tumor engraftment, mice were stratified by flux into treatment groups. Mock or CAR NK-92MI cells (5 × 10⁶) were injected at the same coordinates.

For lymphoma models, female NPG mice were intravenously injected with 5 × 10⁴ Raji-FFLuc or 1 × 10⁵ Nalm-6-FFLuc cells. Four days later, mice received 5 × 10⁶ mock or CAR NK-92MI cells via tail vein injection.

### Statistical methods

Sample sizes were not determined by statistical tests, and researchers were not blinded to experimental groups. Specific statistical tests for determining significance and corresponding P values are provided in the figure legends. All analyses were performed using GraphPad Prism software. P < 0.05 was considered statistically significant.

## Supplementary Material

Supplementary figures.

## References

[1] Park JH, Rivière I, Gonen M, Wang X, Sénéchal B, Curran KJ (2018). Long-term follow-up of CD19 CAR therapy in acute lymphoblastic leukemia. N Engl J Med.

[2] Chung JB, Brudno JN, Borie D, Kochenderfer JN (2024). Chimeric antigen receptor T cell therapy for autoimmune disease. Nat Rev Immunol.

[3] Singh AK, McGuirk JP (2020). CAR T cells: continuation in a revolution of immunotherapy. Lancet Oncol.

[4] Liu E, Marin D, Banerjee P, Macapinlac HA, Thompson P, Basar R (2020). Use of CAR-transduced natural killer cells in CD19-positive lymphoid tumors. N Engl J Med.

[5] Marin D, Li Y, Basar R, Rafei H, Daher M, Dou J (2024). Safety, efficacy and determinants of response of allogeneic CD19-specific CAR-NK cells in CD19(+) B cell tumors: a phase 1/2 trial. Nat Med.

[6] Burger MC, Forster MT, Romanski A, Straßheimer F, Macas J, Zeiner PS (2023). Intracranial injection of NK cells engineered with a HER2-targeted chimeric antigen receptor in patients with recurrent glioblastoma. Neuro Oncol.

[7] Tang X, Yang L, Li Z, Nalin AP, Dai H, Xu T (2018). First-in-man clinical trial of CAR NK-92 cells: safety test of CD33-CAR NK-92 cells in patients with relapsed and refractory acute myeloid leukemia. Am J Cancer Res.

[8] Laskowski TJ, Biederstädt A, Rezvani K (2022). Natural killer cells in antitumour adoptive cell immunotherapy. Nat Rev Cancer.

[9] Shah K, Al-Haidari A, Sun J, Kazi JU (2021). T cell receptor (TCR) signaling in health and disease. Signal Transduct Target Ther.

[10] Li Y, Hermanson DL, Moriarity BS, Kaufman DS (2018). Human iPSC-Derived natural killer cells engineered with chimeric antigen receptors enhance anti-tumor activity. Cell Stem Cell.

[11] Acharya S, Basar R, Daher M, Rafei H, Li P, Uprety N (2024). CD28 costimulation augments CAR signaling in NK cells via the LCK/CD3ζ/ZAP70 signaling axis. Cancer Discov.

[12] Bryceson YT, March ME, Ljunggren HG, Long EO (2006). Activation, coactivation, and costimulation of resting human natural killer cells. Immunol Rev.

[13] Bryceson YT, March ME, Ljunggren HG, Long EO (2006). Synergy among receptors on resting NK cells for the activation of natural cytotoxicity and cytokine secretion. Blood.

[14] Ra C, Jouvin MH, Blank U, Kinet JP (1989). A macrophage Fc gamma receptor and the mast cell receptor for IgE share an identical subunit. Nature.

[15] Wirthmueller U, Kurosaki T, Murakami MS, Ravetch JV (1992). Signal transduction by Fc gamma RIII (CD16) is mediated through the gamma chain. J Exp Med.

[16] Letourneur O, Kennedy IC, Brini AT, Ortaldo JR, O'Shea JJ, Kinet JP (1991). Characterization of the family of dimers associated with Fc receptors (Fc epsilon RI and Fc gamma RIII). J Immunol.

[17] Billadeau DD, Upshaw JL, Schoon RA, Dick CJ, Leibson PJ (2003). NKG2D-DAP10 triggers human NK cell-mediated killing via a Syk-independent regulatory pathway. Nat Immunol.

[18] Fauriat C, Long EO, Ljunggren HG, Bryceson YT (2010). Regulation of human NK-cell cytokine and chemokine production by target cell recognition. Blood.

[19] Bryceson YT, March ME, Barber DF, Ljunggren HG, Long EO (2005). Cytolytic granule polarization and degranulation controlled by different receptors in resting NK cells. J Exp Med.

[20] Das A, Long EO (2010). Lytic granule polarization, rather than degranulation, is the preferred target of inhibitory receptors in NK cells. J Immunol.

[21] Bryceson YT, Ljunggren HG, Long EO (2009). Minimal requirement for induction of natural cytotoxicity and intersection of activation signals by inhibitory receptors. Blood.

[22] Johnston SC, Dustin ML, Hibbs ML, Springer TA (1990). On the species specificity of the interaction of LFA-1 with intercellular adhesion molecules. J Immunol.

[23] Bryceson YT, Long EO (2008). Line of attack: NK cell specificity and integration of signals. Curr Opin Immunol.

[24] Vivier E, Tomasello E, Baratin M, Walzer T, Ugolini S (2008). Functions of natural killer cells. Nat Immunol.

[25] Wei S, Gamero AM, Liu JH, Daulton AA, Valkov NI, Trapani JA (1998). Control of lytic function by mitogen-activated protein kinase/extracellular regulatory kinase 2 (ERK2) in a human natural killer cell line: identification of perforin and granzyme B mobilization by functional ERK2. J Exp Med.

[26] Ottinger EA, Botfield MC, Shoelson SE (1998). Tandem SH2 domains confer high specificity in tyrosine kinase signaling. J Biol Chem.

[27] Kanakaraj P, Duckworth B, Azzoni L, Kamoun M, Cantley LC, Perussia B (1994). Phosphatidylinositol-3 kinase activation induced upon Fc gamma RIIIA-ligand interaction. J Exp Med.

[28] Bonnema JD, Karnitz LM, Schoon RA, Abraham RT, Leibson PJ (1994). Fc receptor stimulation of phosphatidylinositol 3-kinase in natural killer cells is associated with protein kinase C-independent granule release and cell-mediated cytotoxicity. J Exp Med.

[29] Xu X, Chong AS (1996). Vav in natural killer cells is tyrosine phosphorylated upon cross-linking of Fc gamma RIIIA and is constitutively associated with a serine/threonine kinase. Biochem J.

[30] Billadeau DD, Brumbaugh KM, Dick CJ, Schoon RA, Bustelo XR, Leibson PJ (1998). The Vav-Rac1 pathway in cytotoxic lymphocytes regulates the generation of cell-mediated killing. J Exp Med.

[31] Trotta R, Puorro KA, Paroli M, Azzoni L, Abebe B, Eisenlohr LC (1998). Dependence of both spontaneous and antibody-dependent, granule exocytosis-mediated NK cell cytotoxicity on extracellular signal-regulated kinases. J Immunol.

[32] Milella M, Gismondi A, Roncaioli P, Bisogno L, Palmieri G, Frati L (1997). CD16 cross-linking induces both secretory and extracellular signal-regulated kinase (ERK)-dependent cytosolic phospholipase A2 (PLA2) activity in human natural killer cells: involvement of ERK, but not PLA2, in CD16-triggered granule exocytosis. J Immunol.

[33] Ting AT, Karnitz LM, Schoon RA, Abraham RT, Leibson PJ (1992). Fc gamma receptor activation induces the tyrosine phosphorylation of both phospholipase C (PLC)-gamma 1 and PLC-gamma 2 in natural killer cells. J Exp Med.

[34] Jevremovic D, Billadeau DD, Schoon RA, Dick CJ, Irvin BJ, Zhang W (1999). Cutting edge: a role for the adaptor protein LAT in human NK cell-mediated cytotoxicity. J Immunol.

[35] Shresta S, MacIvor DM, Heusel JW, Russell JH, Ley TJ (1995). Natural killer and lymphokine-activated killer cells require granzyme B for the rapid induction of apoptosis in susceptible target cells. Proc Natl Acad Sci U S A.

[36] Lowin B, Beermann F, Schmidt A, Tschopp J (1994). A null mutation in the perforin gene impairs cytolytic T lymphocyte- and natural killer cell-mediated cytotoxicity. Proc Natl Acad Sci U S A.

[37] Upshaw JL, Arneson LN, Schoon RA, Dick CJ, Billadeau DD, Leibson PJ (2006). NKG2D-mediated signaling requires a DAP10-bound Grb2-Vav1 intermediate and phosphatidylinositol-3-kinase in human natural killer cells. Nat Immunol.

[38] Upshaw JL, Schoon RA, Dick CJ, Billadeau DD, Leibson PJ (2005). The isoforms of phospholipase C-gamma are differentially used by distinct human NK activating receptors. J Immunol.

[39] Galea-Lauri J, Darling D, Gan SU, Krivochtchapov L, Kuiper M, Gäken J (1999). Expression of a variant of CD28 on a subpopulation of human NK cells: implications for B7-mediated stimulation of NK cells. J Immunol.

[40] Azuma M, Cayabyab M, Buck D, Phillips JH, Lanier LL (1992). Involvement of CD28 in MHC-unrestricted cytotoxicity mediated by a human natural killer leukemia cell line. J Immunol.

