# Supplementary material for: Natural killer cell-specific chimeric antigen receptor enhances CAR NK cell functions and anti-tumor activity
Source: Theranostics. 2025 Nov 6;15(17):9344–58. doi: 10.7150/thno.120909 (PMC12630554; doi:10.7150/thno.120909)
Supplement: Supplementary file 1 — Supplementary figures. [file thnov15p9344s1.pdf]

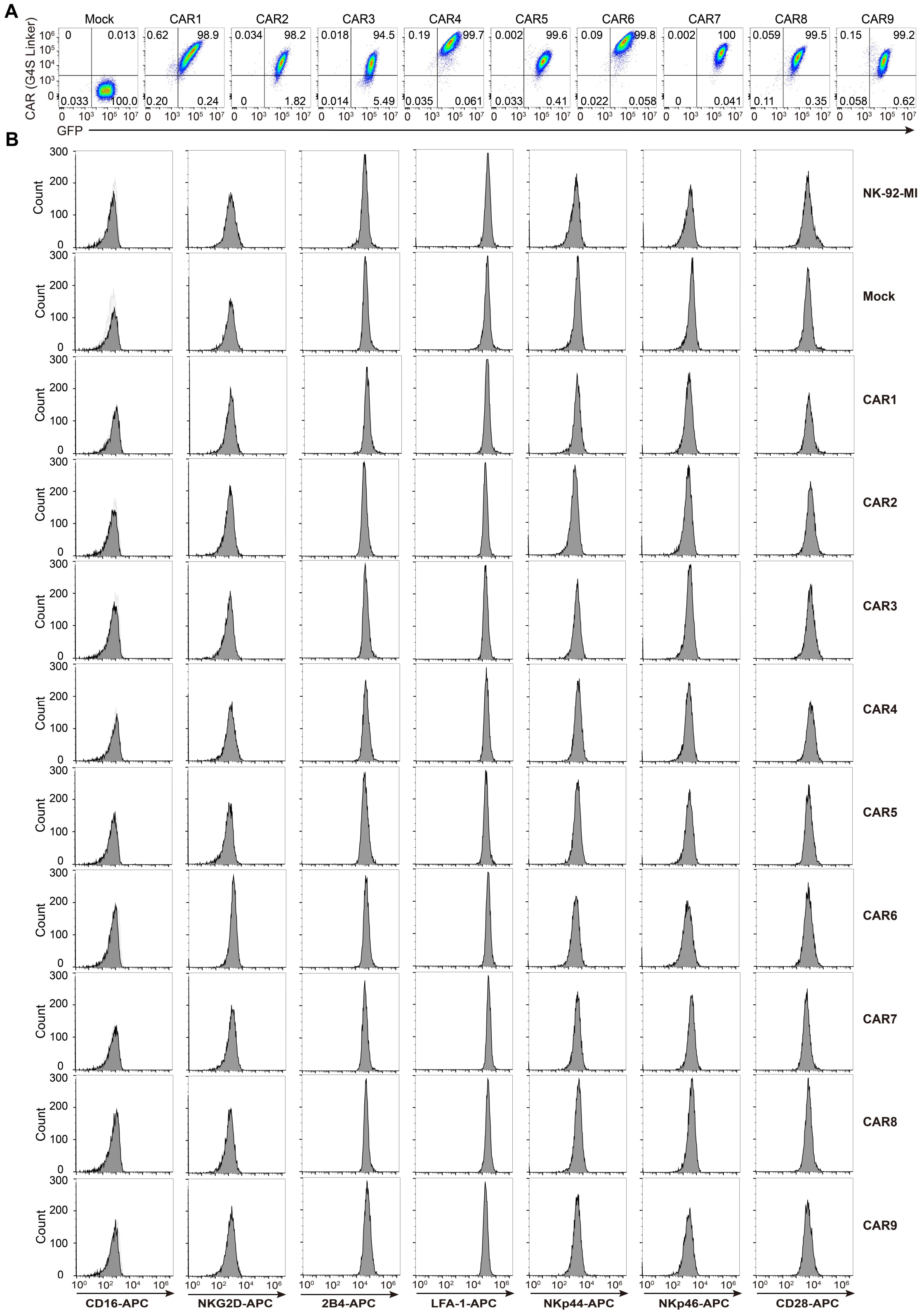

Figure S1. Expression of CAR and endogeneous activating receptors in NK-92MI cells.

(**A**) Expression of GFP and CAR molecules in NK-92MI cells as assessed by flow cytometry. (**B**) Flow cytometric analysis of major NK cell activating receptors, including CD16, NKG2D, 2B4, LFA-1, NKp44, NKp46, and CD28.

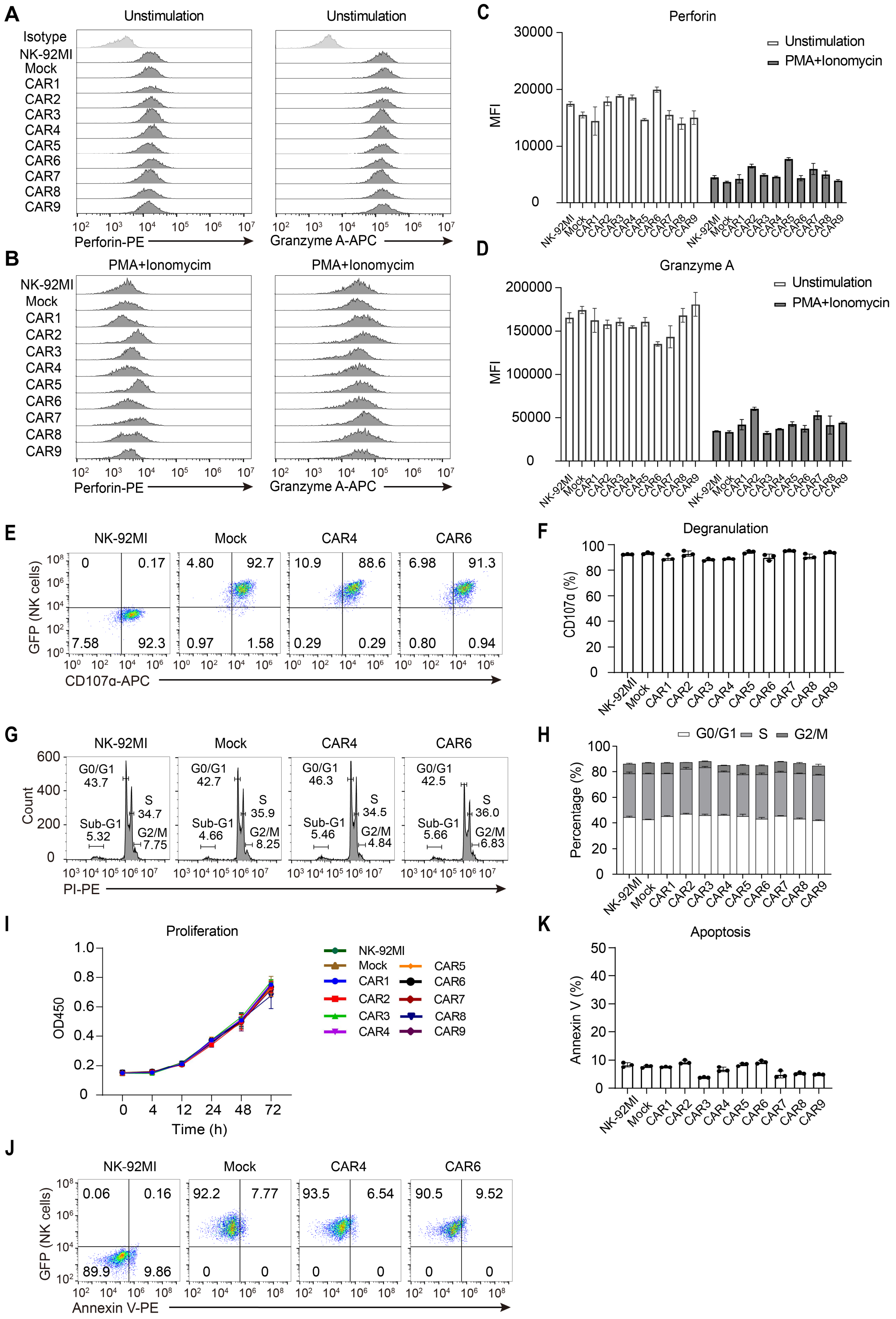

Figure S2. Cellular functions of CAR NK-92MI cells following CAR molecule modification.

(**A-B**) Representative intracellular flow cytometry staining of perforin and granzyme A without stimulation (**A**) or stimulation by PMA and ionomycin (**B**). (**C-D**) Summary of perforin (**C**) and

granzyme A (**D**) expression before and after PMA and ionomycin stimulation (n = 3). (**E**) Representative degranulation assay using CD107α expression following stimulation with PMA and ionomycin. (**F**) Summary of degranulation (n = 3). (**G**) Representative flow cytometric analysis of the cell cycle. (**H**) Cell cycle distribution of the indicated CAR NK-92MI cell lines (n = 3). (**I**) Proliferation was assessed using the CCK-8 method over a 3-day culture period (n = 3). (**J**) Representative flow cytometric analysis of cell apoptosis. (**K**) Quantification of cell apoptosis by identifying annexin V-positive cells (n = 3).

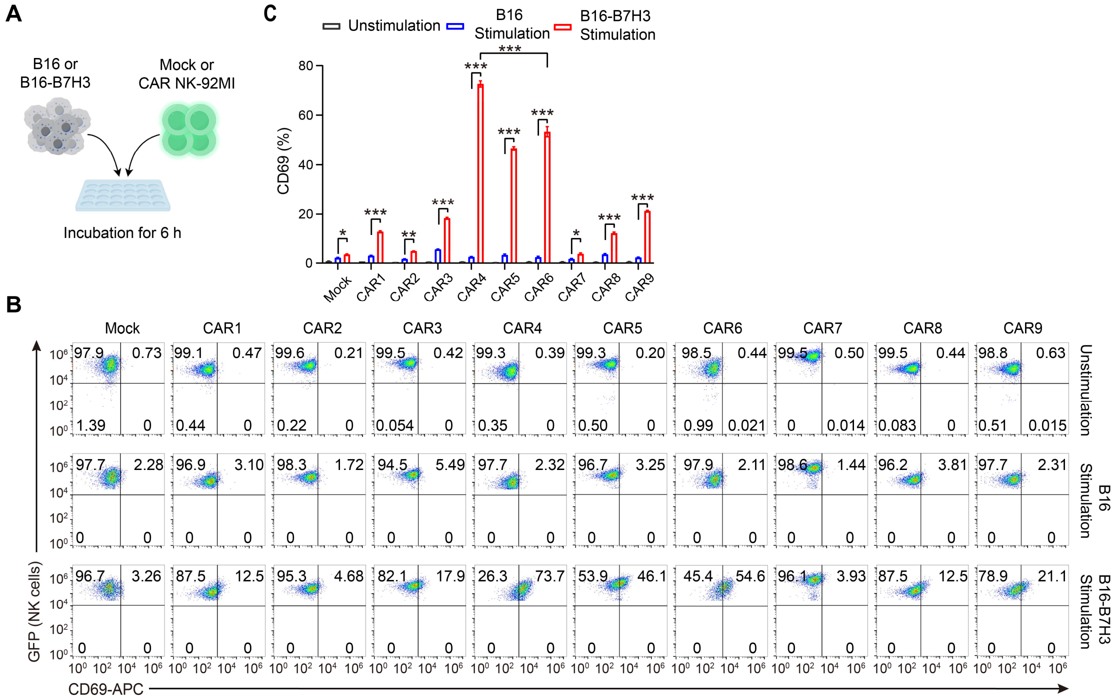

Figure S3. Specific induction of CAR NK cell activation by B16-B7H3 cells.

(**A**) Schematic illustration of NK cell activation assay, where CD69 expression was evaluated after 6 h coculture with B16 or B16-B7H3 cells. (**B**) Representative flow cytometric results of CD69 expression. (**C**) Summary of CD69 expression. (n = 3). Data are presented as mean ± s.d. Statistical analysis was performed using two-way ANOVA followed by Tukey's multiple comparisons test.

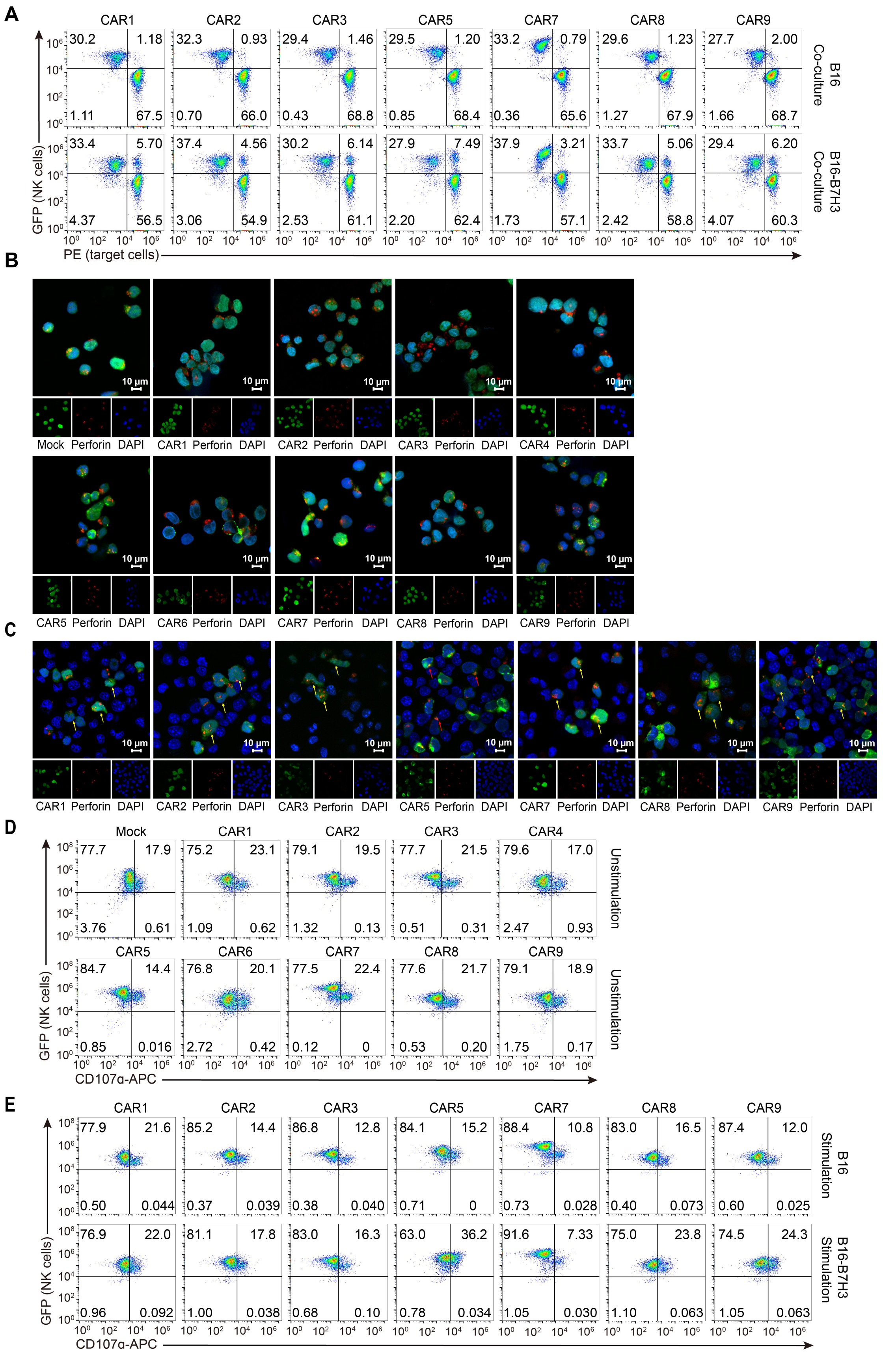
A
CAR1 CAR2 CAR3 CAR5 CAR7 CAR8 CAR9
B16 Co-culture
B16-B7H3 Co-culture
GFP (NK cells)
PE (target cells)
B
Mock Perforin DAPI
CAR1 Perforin DAPI
CAR2 Perforin DAPI
CAR3 Perforin DAPI
CAR4 Perforin DAPI
CAR5 Perforin DAPI
CAR6 Perforin DAPI
CAR7 Perforin DAPI
CAR8 Perforin DAPI
CAR9 Perforin DAPI
10 μm
C
CAR1 Perforin DAPI
CAR2 Perforin DAPI
CAR3 Perforin DAPI
CAR5 Perforin DAPI
CAR7 Perforin DAPI
CAR8 Perforin DAPI
CAR9 Perforin DAPI
D
Mock CAR1 CAR2 CAR3 CAR4
CAR5 CAR6 CAR7 CAR8 CAR9
Unstimulation
GFP (NK cells)
CD107a-APC
E
CAR1 CAR2 CAR3 CAR5 CAR7 CAR8 CAR9
B16 Stimulation
B16-B7H3 Stimulation
GFP (NK cells)
CD107a-APC

Figure S4. In vitro characterization of NK cell-specific CAR constructs in NK 92-MI cells.

(**A**) Representative conjugate formation of CAR1-3, CAR5, and CAR7-9 NK-92MI cells. NK cell-target cell conjugates were identified as $PE^{+}GFP^{+}$ cells in the upper right quadrant. (**B**) Representative confocal microscopy images of GFP-expressing Mock and CAR NK-92MI cells under unstimulated conditions, stained with Alexa 594-conjugated anti-perforin monoclonal antibody (mAb) using a 60× oil immersion objective. (**C**) Representative confocal microscopy images of GFP-expressing CAR1-3, CAR5, and CAR7-9 NK cells cocultured with B16-B7H3 cells, stained with Alexa 594-conjugated anti-perforin mAb. Perforin-containing granules were considered polarized when most randomly distributed fluorescence was concentrated in a quadrant of the NK-92MI cell. Red and yellow arrows indicate polarized and non-polarized granules, respectively. (**D**) Representative flow cytometric results of degranulation, assessed by CD107α expression, in unstimulated Mock and CAR NK-92MI cells. (**E**) Representative degranulation of CAR1-3, CAR5, and CAR7-9 NK-92MI cells against B16 or B16-B7H3 target cells.

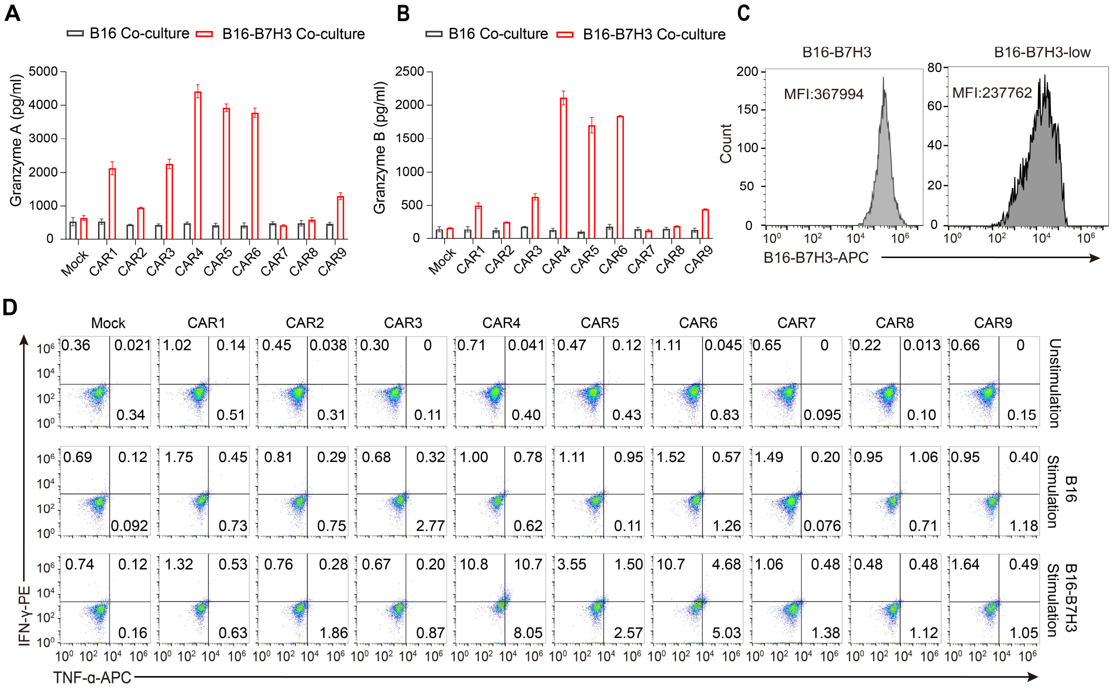

Figure S5. Cytokine production and B16-B7H3-low cell line construction.

(**A-B**) Mock or CAR NK-92MI cells (1×10^6) were cocultured with target cells (1×10^6) for 6 h. Supernatants were collected, and granzyme A (**A**) and granzyme B (**B**) levels were measured using ELISA kits. (**C**) Following staining of B16-B7H3 cells with anti-B7H3 antibody, the subpopulation with the lowest B7H3 expression (accounting for ~1% of total cells) was isolated via flow sorting to generate the B16-B7H3-low cell line. (**D**) Representative flow cytometric results of intracellular IFN-γ and TNF-α expression. IFN-γ expression was quantified as the sum of cells in the first and second quadrants; TNF-α expression was quantified as the sum of cells in the first and fourth quadrants.

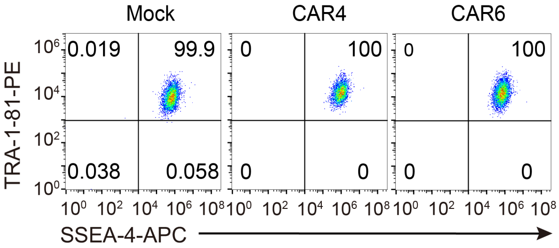

Figure S6. Flow cytometric analysis of stemness in iPSCs, as marked by SSEA-4 and TRA-1-81.

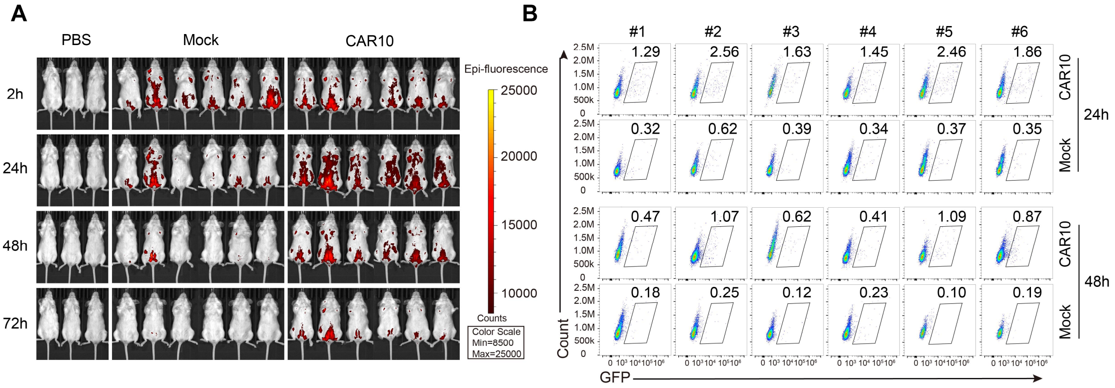

Figure S7. Biodistribution and persistence of CAR NK-92MI cells in Raji murine model.

(**A**) NPG mouse xenografts were established with luciferase (luc)-expressing Raji cells and treated with Mock or CAR10 NK-92MI cells as depicted in figure 6 (n = 3 or 6 per group). CAR NK cells were engineered to express green fluorescent protein (GFP) during construction. Therefore, Mock or CAR10 NK-92MI cells were monitored via fluorescence using the IVIS Spectrum In Vivo Imaging System (PerkinElmer) at the indicated time points after NK cell infusion. Given the strong autofluorescence from mouse fur and other tissues, tumor-bearing mice without NK cell injection were used as controls to subtract autofluorescence background. (**B**) Pharmacokinetic profiles of Mock or CAR10 NK-92MI cells. At specified time points, venous blood was collected, and the proportion of GFP-positive cells in peripheral blood was analyzed by flow cytometry.
